# Homocysteine Editing, Thioester Chemistry, Coenzyme A, and the Origin of Coded Peptide Synthesis †

**DOI:** 10.3390/life7010006

**Published:** 2017-02-09

**Authors:** Hieronim Jakubowski

**Affiliations:** 1 Department of Microbiology, Biochemistry and Molecular Genetics, New Jersey Medical School, Rutgers University, Newark, NJ 07103, USA; jakubows2@gmail.com or jakubows@rutgers.edu; Tel.: +1-973-972-8733; 2 Department of Biochemistry and Biotechnology, University of Life Sciences, Poznan 60-632, Poland

**Keywords:** aminoacyl-tRNA synthetase, homocysteine editing, thioester, coenzyme A, non-coded peptide synthesis, prebiotic chemistry, thioester world, evolution

## Abstract

Aminoacyl-tRNA synthetases (AARSs) have evolved “quality control” mechanisms which prevent tRNA aminoacylation with non-protein amino acids, such as homocysteine, homoserine, and ornithine, and thus their access to the Genetic Code. Of the ten AARSs that possess editing function, five edit homocysteine: Class I MetRS, ValRS, IleRS, LeuRS, and Class II LysRS. Studies of their editing function reveal that catalytic modules of these AARSs have a thiol-binding site that confers the ability to catalyze the aminoacylation of coenzyme A, pantetheine, and other thiols. Other AARSs also catalyze aminoacyl-thioester synthesis. Amino acid selectivity of AARSs in the aminoacyl thioesters formation reaction is relaxed, characteristic of primitive amino acid activation systems that may have originated in the Thioester World. With homocysteine and cysteine as thiol substrates, AARSs support peptide bond synthesis. Evolutionary origin of these activities is revealed by genomic comparisons, which show that AARSs are structurally related to proteins involved in coenzyme A/sulfur metabolism and non-coded peptide bond synthesis. These findings suggest that the extant AARSs descended from ancestral forms that were involved in non-coded Thioester-dependent peptide synthesis, functionally similar to the present-day non-ribosomal peptide synthetases.

## 1. Introduction

**Figure 1:**
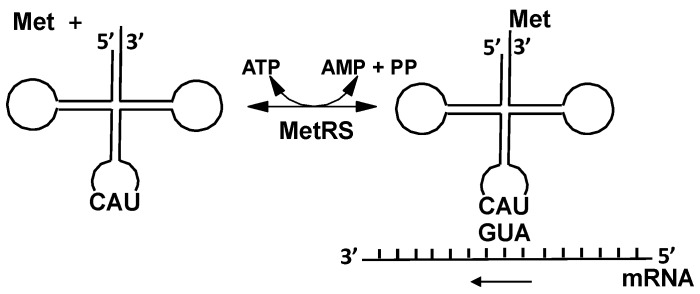
Chemical activation and information transfer by aminoacyl-tRNA synthetases (AARSs).

Each of the 20 aminoacyl-tRNA synthetases (AARSs) fulfils two important functions in the initial steps in the translation of the Genetic Code: Chemical Activation and Information Transfer. For example, methionyl-tRNA synthetase (MetRS) catalyzes Chemical Activation of the carboxyl group of its cognate amino acid methionine using ATP, which affords Met-AMP bound to the catalytic module of MetRS (Figure 1). The Information Transfer function involves attachment of the activated Met to the 3′ adenosine of tRNA(CAU)^Met^ according to the rules of the Genetic Code thereby matching Met with its anticodon CAU, which is read by the AUG codon in the mRNA on the ribosome (Figure 1).

AARSs belong to two structurally unrelated classes, of ten enzymes each, which have different catalytic domains indicating their independent evolutionary origin [1,2]. Class I AARSs usually have monomeric structure with a Rossman-fold catalytic domain characterized by the HIGH and KMSKS signature sequences. Class II AARSs have a two- or four-subunit quaternary structure and an antiparallel β sheet catalytic domain with class II-defining motifs. With the exception of LysRS enzymes, which exist as a Class I or Class II structure in different organisms, other AARSs have a class-specific structure in the three domains of life.

Catalytic domains of Class I and II AARSs exhibit pronounced differences in their modes of substrate binding. For example, class I AARSs bind ATP in an extended conformation, while class II AARSs bind ATP in a bent conformation with the γ-phosphate folding back over the adenine ring. While Class I AARSs bind tRNA via the minor groove side of its amino acid acceptor stem helix, Class II AARSs bind tRNA via the major groove side. Catalytic domains of Class I and II AARSs exhibit also *functional* differences. Specifically, Class II AARS, such as LysRS, PheRS, HisRS, SerRS, and AspRS, catalyze formation of diadenosine 5′,5′′′-*P*^1^,*P*^4^-tetraphosphate (AppppA) [3,4,5], a signaling molecule that participates in transcriptional regulation of IgE-mediated immune response [6,7]. In contrast, Class I AARSs, such as ArgRS and TrpRS, do not possess the AppppA synthetase activity or have >10–100-fold lower activity (ValRS, MetRS, TyrRS) [4,5,8].

AARSs exhibit high selectivity for their cognate amino acid and tRNA substrates with error rates in the section of amino acids and tRNAs of 10^−4^ to 10^−5^ and 10^−6^, respectively [9,10,11,12]. Although in general unambiguous translation according to the rules of the Genetic Code is crucial for cellular homeostasis, some species have adapted to grow optimally in the presence of ambiguous translation since, under stress conditions, higher error rates assure survival [13,14,15]. AARSs achieve unambiguous pairing of amino acids with their cognate tRNAs by preferential binding of cognate amino acids and a *quality control* [16] step, in which non-cognate amino acids are selectively edited [10,11,16,17]. The *quality control* step involves either *pre-transfer* or *post-transfer* mechanisms, or both [18]. The major *pre-transfer* mechanism involves hydrolysis of AA~AMP at the catalytic domain, first discovered for ValRS, IleRS, and MetRS [18,19], while *post-transfer* mechanism involves hydrolysis of AA-tRNA at a separate editing domain, originally discovered for IleRS [20] and PheRS [21]. Of the 20 extant AARSs, ten possess an editing function which corrects errors in amino acid selection [22]. For some AARS (IleRS, ValRS, or AlaRS), the editing function is conserved throughout the three domains of life, while editing function of other AARSs (LeuRS, ProRS, or PheRS) is phylogenetically restricted ([23] and references therein).

Editing by AARSs prevents access of *non-proteinogenic* amino acids such as homocysteine [24,25,26,27,28], ornithine [29], homoserine [10,16], or norvaline [30] to the Genetic Code and effectively partitions amino acids present in extant organisms into *proteinogenic* and *non-proteinogenic* amino acids. Natural non-proteinogenic amino acids vastly outnumber the 20 canonical proteinogenic amino acids found in all organisms, plus selenocysteine and pyrrolysine encoded in only some genomes [31]. Hundreds of non-proteinogenic amino acids are known in various species [32]: about 240 in plants, 75 in fungi, 50 in animals, and 50 in prokaryotes [33].

Of the 10 AARS that possess the editing function, five edit the thiol amino acid homocysteine (Hcy) at the catalytic domain: Class I MetRS, LeuRS, IleRS, ValRS, and Class II LysRS [10,11]. Other misactivated amino acids are edited at the catalytic domain, a dedicated editing domain, or both [11,31]. Phylogenetic analyses of structural domains present in proteomes [34] suggest that catalytic domains of AARSs belong to the oldest fold families and may have appeared about 3.7 billion years ago, while separate domains that edit misacylated tRNA appeared later, about 3.2 billion years ago [35] (these timelines are based on counting relative node, i.e., branch point, distance in the rooted trees and using a molecular clock of protein folds to convert relative age into geological time [36]).

Because of their crucial role in the translation and maintenance of the Genetic Code, analysis of AARSs structures and mechanisms of reactions catalyzed by AARSs can provide insights into the origin and evolution of the Genetic Code [35,37]. The present article examines the links between Hcy editing, thioester chemistry, and the origin of the amino acid activation for the coded protein synthesis. Available data suggest that ancestral AARSs were involved in the thiol (coenzyme A, pantetheine) aminoacylation reactions and thioester-based non-coded peptide synthesis before the emergence of the Genetic Code and the ribosomal protein biosynthetic machinery.

## 2. Homocysteine (Hcy) is Edited by Class I and Class II Aminoacyl-tRNA Synthetases (AARSs)

One of the selectivity problems in protein biosynthesis is discrimination against the non-proteinogenic thiol amino acid Hcy, a universal precursor of methionine. Hcy is misactivated (Reaction (1)) by Class I MetRS, IleRS, LeuRS [25,26], ValRS [18] and class II LysRS [10,11,29].

AARS + Hcy + ATP **⇄** AARS•Hcy~AMP + PP_i_ (1)

Misactivated Hcy is edited by an intramolecular reaction between the side chain thiolate and the activated carboxyl of Hcy, affording the thioester Hcy-thiolactone (Reaction (2)) [18,38].

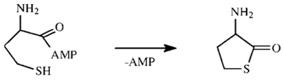
(2)

Hcy editing does not depend on tRNA [10,18], consumes one mole of ATP per mole Hcy-thiolactone [38], and prevents attachment of Hcy to tRNA, and thus Hcy access to the Genetic Code.

The energy of the anhydride bond of Hcy~AMP is conserved in the thioester bond of Hcy-thiolactone. Consequently, Hcy-thiolactone easily reacts with free amino acids forming Hcy-AA dipeptides [39,40] and with protein lysine residues forming *N*-Hcy-protein [39,40,41].

### 2.1. Hcy Editing is Universal

Hcy is an important intermediate in the metabolism of Met, Cys, and one-carbon units carried on folates in *Bacteria* and *Eukarya* [41,42]. Hcy has also been shown to be an intermediate in Met and Cys metabolism in *Archaea* methanogens [43]. Because Hcy is a non-coded amino acid, living organisms must have evolved the ability to prevent its access to the Genetic Code. Indeed, in bacteria (*E. coli*, *M. smegmatis*) [24,44], the yeast *Saccharomyces cerevisiae* [27], plants [45], mice [28], and humans [28,46], Hcy is edited and metabolized to Hcy-thiolactone by MetRS. In *E. coli* and *S. cerevisiae* Hcy thiolactone accumulation is proportional to the expression level of MetRS. In *S. cerevisiae*, both cytoplasmic and mitochondrial MetRSs edit Hcy [47]. Editing of endogenous Hcy by MetRS in cultured microbial and mammalian cells is prevented by supplementation with excess Met. In *E. coli* cultures supplemented with Hcy, two other AARSs LeuRS and IleRS, in addition to MetRS, catalyze Hcy-thiolactone formation [25,26]. As a result, Hcy-thiolactone formation is fully prevented only by simultaneous supplementation with excess Ile, Leu, and Met.

In all organisms, including human, genetic deficiencies in the Hcy/Cys/Met pathways or inadequate supply of cofactors of enzymes participating in Hcy metabolism (folate, cobalamin/vitamin B_12_, pyridoxal phosphate/vitamin B_6_) lead to the accumulation of Hcy and its metabolites, including Hcy-thiolactone, which are implicated in cardiovascular and neurodegenerative diseases [41] through mechanisms involving pro-atherogenic changes in gene expression [48], modification of protein structure [40] leading to amyloid formation [49], activation of mTORC1 signaling and inhibition of autophagy [50], and induction of inflammatory and autoimmune responses [51,52,53].

Structural similarities between Archaeal and Bacterial MetRSs suggest that Hcy can also be edited by Archaeal MetRSs. For example the catalytic domain of the Archeon *Pyrococcus abyssi* MetRS is very similar to catalytic domains of *E. coli* and *T. thermophilus* MetRSs and can be superimposed with a root mean square deviation (RMSD) values of 1.7 Å for 481 Cα atoms and 1.6 Å for 406 Cα atoms, respectively. Residues important for the synthetic and editing functions of the Bacterial MetRSs are conserved in the Archaeal MetRS and have similar positions in crystal structures [54], including a glutamic acid residue, E259, in *P. abyssi* MetRS homologous to aspartic acid D259 residue in *E. coli* MetRS, which participates as a mechanistic base in the Hcy editing reaction (discussed in Section 2.2.1. below).

### 2.2. Mechanism of Hcy Editing

Hcy editing is unique in that it involves an *intramolecular* Reaction (2), in which the side chain thiolate of Hcy molecule is a nucleophile, to accomplish editing. Editing reactions of all other amino acids, including a related thio-amino acid Cys, are *intermolecular* and use water hydroxide as a nucleophile [11]. Hcy is edited by the cyclization to Hcy-thiolactone at the synthetic/editing catalytic site in the Rossman-fold domain of class I AARS [55,56] and at the β sheet catalytic domain of Class II LysRS [29,38].

#### 2.2.1. Methionyl-tRNA Synthetase (MetRS)

A model for pre-transfer Hcy editing explains how MetRS partitions Met and Hcy between the synthetic and editing pathways, respectively. The model is supported by the crystal structure [57], structure/function [55,56], and computational [58] studies of *E. coli* MetRS. In the synthetic pathway, the activated carboxyl of Met reacts with the 2′-hydroxyl of the 3′-terminus of tRNAMet, affording Met-tRNAMet. In the editing pathway, the activated carboxyl of Hcy reacts with the thiolate of its side chain, affording Hcy~thiolactone.

**Figure 2:**
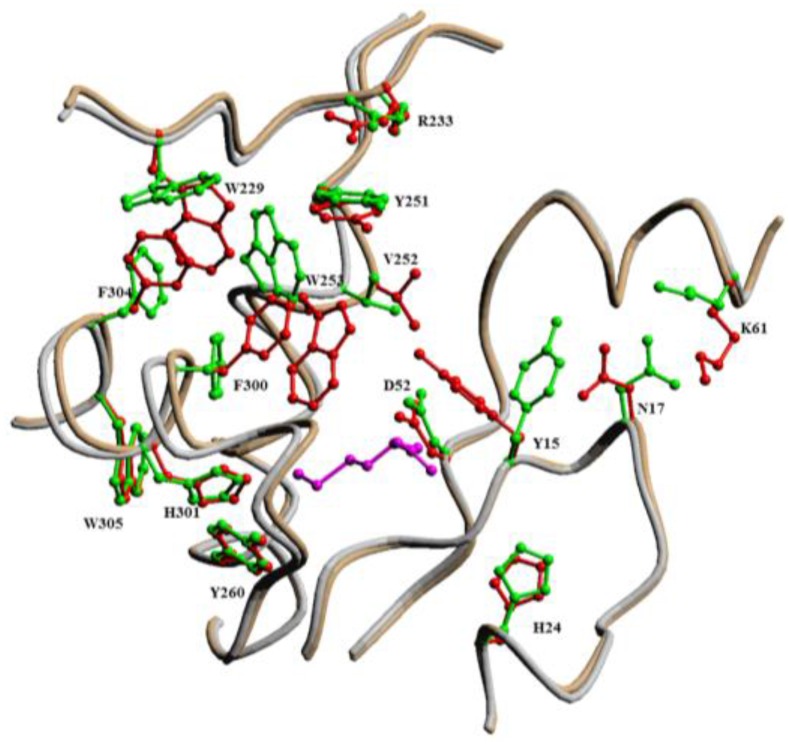
The synthetic/editing active site of *E. coli* methionyl-tRNA synthetase (MetRS): Hydrophobic and hydrogen-bonding interactions provide specificity for the cognate substrate l-methionine. Superimposition of Cα carbon atoms for the MetRS·Met complex (beige) and free MetRS (light grey), solved at 1.8 Å resolution, shows movements of active site residues upon binding of Met. Residue colors are red in the MetRS·Met complex and green in free MetRS, and l-methionine is magenta. Reprinted with permission from ref. [57].

Whether an amino acid substrate completes the synthetic or editing pathway is determined by the partitioning of its side chain between the specificity and thiol-binding subsites [55]. Met completes the synthetic pathway because its side chain is bound by the hydrophobic and hydrogen-bonding interactions with W305 and Y15 residues in the specificity sub-site (Figure 2).

**Figure 3:**
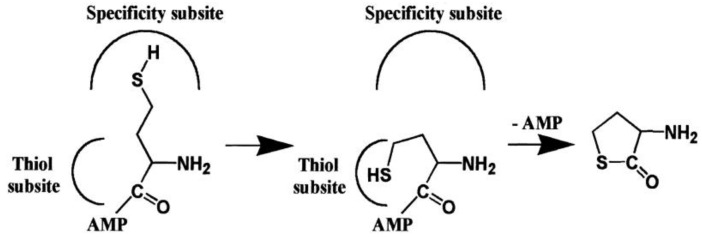
Editing of miscativated homocysteine (Hcy~AMP) at the catalytic module of an AARS: The MetRS-catalyzed cyclization of homocysteinyl adenylate to form Hcy-thiolactone and AMP, which are subsequently released from the synthetic/editing active site of MetRS.

In contrast, the side chain of Hcy, missing the methyl group of Met, interacts weakly with the specificity sub-site. This allows the side chain of Hcy to interact with D259 [58] in the thiol-binding sub-site [55], which facilitates editing by cyclization to Hcy-thiolactone (Figure 3). Consistent with this model, mutations of W305 and Y15 residues, which form the hydrophobic Met-binding sub-site, reduce the Hcy/Met discrimination by the enzyme [56].

**Figure 4:**
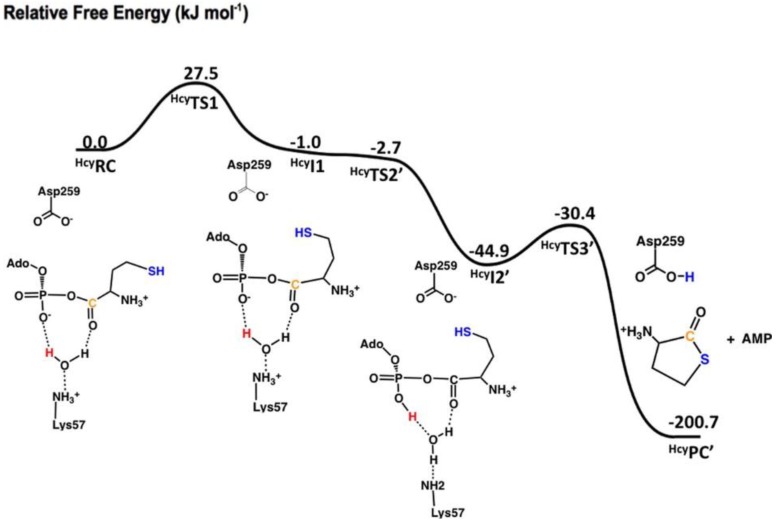
Energetics of Hcy~AMP editing at the catalytic module of MetRS. Asp259 is a mechanistic base that deprotonates the side chain thiol in Hcy~AMP. Reproduced with permission from ref. [58].

Computational studies [58] suggest that D259 plays an essential role as a mechanistic base that deprotonates the side chain thiol in Hcy~AMP at the catalytic module of MetRS (Figure 4). In the initial MetRS·Hcy~AMP complex the distance S_Hcy_···O_Asp259_ (5.30 Å) is markedly shorter than the distance S_Hcy_···O_phos_ in Hcy~AMP (7.15 Å) (Table 1). The rate-limiting step in Hcy-thiolactone formation is the rotation about the C_β_−C_γ_ bond with 27.5 kJ·mol^−1^ energy barrier. This is more favorable than 98.25 kJ·mol^−1^ energy barrier for an alternative substrate-assisted mechanism in which the non-bridging oxygen of phosphate in Hcy~AMP acts as a base.

**Figure 5:**
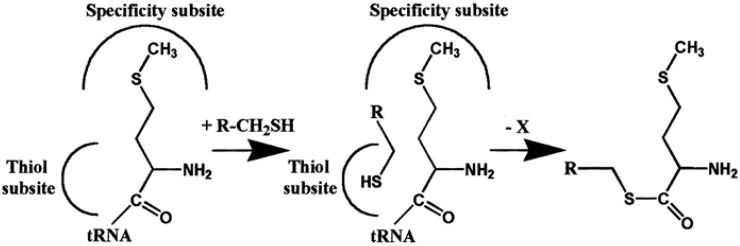
Amino acid:coenzyme A (CoA-SH) ligase activity of MetRS. When the active site is occupied by Met-tRNA or Met-AMP, and the thiol subsite is occupied by CoA-SH, MetRS catalyzes the formation of Met-*S*-CoA thioester. An R represents the bulk of CoA-SH or other thiol molecule.

Cognate Met can also enter the editing pathway when the thiol-binding subsite is occupied by a thiol mimicking the side chain of Hcy [55]. Under these circumstances the activated carboxyl and thiol functions are on separate molecules and MetRS becomes a Met:thiol ligase that catalyzes synthesis of Met thioesters (Figure 5).

#### 2.2.2. Leucyl-tRNA Synthetase (LeuRS), Isoleucyl-tRNA Synthetase (IleRS), Valyl-tRNA Synthetase (ValRS)

A similar model explains Hcy editing [25,26] by related Class I AARS. ValRS, LeuRS, and IleRS have active sites, D490, E532, and E550, respectively, that correspond to D359 of MetRS and are similarly positioned with respect to the C_carb_ center of the substrate Hcy~AMP [58]. Computational analyses show that the Hcy~AMP substrate binds in their active sites in a linear conformation similar to that observed for MetRS. The average C_carb_···O_Asp/Glu_ distances are within 1.16 Å of each other whereas all S_Hcy_···C_carb_ distances are within 1.24 Å of each other (Table 1). More importantly, for MetRS, LeuRS, and ValRS, the average S_Hcy_···O_Asp/Glu_ distance is significantly shorter than the average S_Hcy_···O_phos_ distance by 2.15 Å, 2.27 Å, and 2.06 Å, respectively. Thus, each of these Asp/Glu residues can act as a mechanistic base that deprotonates the side chain thiol in Hcy~AMP and facilitate Hcy-thiolactone formation by MetRS, LeuRS, and ValRS. However, it is less clear whether Glu550 can act as a base in Hcy-thiolactone formation catalyzed by IleRS.

#### 2.2.3. Lysyl-tRNA Synthetase (LysRS) Edits Homocysteine (Hcy), Ornithine (Orn), Homoserine (Hse), but Mischarges tRNA^Lys^ with Proteinogenic Amino Acids

Hcy editing occurs also at the β sheet catalytic domain of Class II *E. coli* LysRS [38], which also edits Orn and Hse. Editing by LysRS is not affected by tRNA^Lys^ and there is no tRNA^Lys^ mischarging with Hcy or Orn [29]. However, LysRS mischarges tRNA^Lys^ with several other amino acids (Arg, Thr, Met, Cys, Leu, Ala, or Ser) and does not deacylate mischarged Arg-tRNA^Lys^, Thr-tRNA^Lys^, and Met-tRNA^Lys^. Recent data show that Met-tRNA^Lys^ (and other tRNAs mischarged with Met) are formed in *E. coli* and mammalian cells in response to stress conditions [13,14].

## 3. Expanding the Genetic Code: Decoding Methionine Codons by Homocysteine

**Figure 6:**
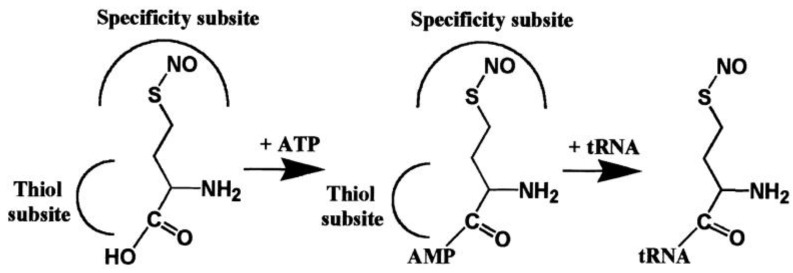
Aminoacylation of tRNA with *S*-NO-Hcy catalyzed by MetRS [59].

By preventing the attachment of Hcy to tRNA, the editing reaction assures that Hcy is excluded from the Genetic Code. Weak interactions of the side chain of Hcy with the specificity subsite allow binding of the Hcy side chain to the thiol-binding subsite in the catalytic domain of MetRS [55,56]. Thus, modifications of the side chain of Hcy that increase binding to the specificity subsite should prevent editing and facilitate the transfer to tRNA^Met^. This is achieved by *S*-nitrosylation of Hcy to *S*-nitroso-Hcy (*S*-NO-Hcy), which binds to the MetRS with affinity 10-fold greater than Hcy and is activated by MetRS to form *S*-NO-Hcy~AMP [59]. In contrast to Hcy-AMP which is edited, *S*-NO-Hcy~AMP is resistant to editing due to stronger binding to the specificity subsite which leads to the transfer of *S*-NO-Hcy to tRNA^Met^, affording *S*-NO-Hcy~tRNA^Met^ (Figure 6).

The *S*-NO-Hcy-tRNA^Met^ has a similar susceptibility to spontaneous deacylation as Met~tRNA^Met^ with a half-life of 28 min. De-nitrosylation of *S*-nitroso-Hcy~tRNA^Met^ affords Hcy~tRNA^Met^, the least stable aminoacyl-tRNA known, which spontaneously deacylates with a half-life of 15 s to form Hcy-thiolactone and free tRNA^Met^.

As expected, *S*-NO-Hcy~tRNA^Met^ is a substrate for protein synthesis on ribosomes, which allows translational incorporation of *S*-NO-Hcy into protein at positions normally occupied by Met [59]. For example, when cultures of *E. coli metE* mutant cells (unable to metabolize Hcy to Met) expressing mouse dihydrofolate reductase (DHFR) protein were supplemented with *S*-NO-Hcy, the DHFR protein was found to contain Hcy. Control experiments in which *E. coli metE* cultures were supplemented with Hcy or Hcy-thiolactone, instead of *S*-NO-Hcy, show that there is no incorporation of Hcy into bacterial proteins [59]. Globin and luciferase, produced in an in vitro mRNA-programmed rabbit reticulocyte protein synthesis system supplemented with *S*-NO-Hcy~tRNA^Met^ contain Hcy at positions normally occupied by Met.

Translationally incorporated Hcy has also been identified in protein from cultured human vascular endothelial cells (HUVECs) (Table 2), which endogenously produce nitric oxide and *S*-nitroso-Hcy [60]. Translationally incorporated Hcy is resistant to Edman degradation whereas post-translationally incorporated Hcy (by the reaction of Hcy-thiolactone with protein lysine residues [40]) is not (Table 2, last row) last, which allows to distinguish between the two mechanisms [61].

Taken together, these findings show that Hcy can gain an access to the Genetic Code by nitric oxide-mediated invasion of the methionine-coding pathway [59].

## 4. AARSs Support the Aminoacylation of Thiols and Peptide Bond Synthesis

### 4.1. Methionyl-tRNA Synthetase (MetRS)

In the model of *pre-transfer* Hcy editing by MetRS, the side chain thiol of Hcy-AMP is bound to D259 at the thiol-binding subsite (Figure 4). When the cognate Met occupies the catalytic site of MetRS, the thiol-binding site is vacant. A prediction of this model is that activated Met (i.e., Met~tRNA or Met~AMP), which binds in the synthetic mode, will enter the editing pathway when the thiol-binding sub-site is filled by a thiol mimicking the side chain of Hcy (Figure 5).

**Scheme 1:**
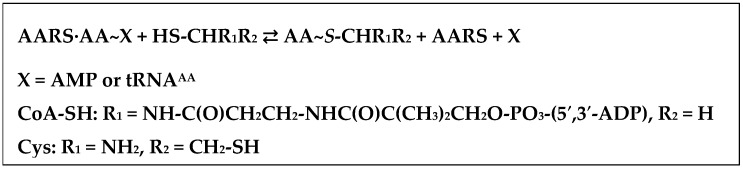
Aminoacylation of thiols catalyzed by an AARS.

This prediction is confirmed by findings showing that MetRS has the ability to catalyze the transfer of Met from Met~AMP or Met~tRNA to a variety of thiols with the formation of Met thioesters [55,63], i.e., has a Met:CoA-SH ligase activity (Scheme 1).

The thiol-binding site exhibits a remarkable selectivity for coenzyme A (CoA-SH) and cysteine, which are the preferred thiol substrates forming Met~*S*-CoA thioester and MetCys dipeptide, respectively. The formation of thioesters and dipeptides exhibits saturation kinetics with respect to thiols, characteristic of enzymatic reactions. Rates of the thiol aminoacylation reaction approach the rate of Hcy editing by MetRS (*k*_cat_ = 1 s^−1^). The rate enhancement by MetRS of the aminoacyl thioester formation reaction is up to 1,000,000-fold [55]. Pantetheine is a 22-fold less catalytically efficient substrate than CoA-SH, indicating that the adenine nucleotide portion of CoA-SH structure is important for the Met~*S*-CoA thioester formation. DesulfoCoA, a CoA-SH analogue without the thiol, is not aminoacylated, indicating absolute requirement of the CoA-SH thiol [63].

**Scheme 2:**
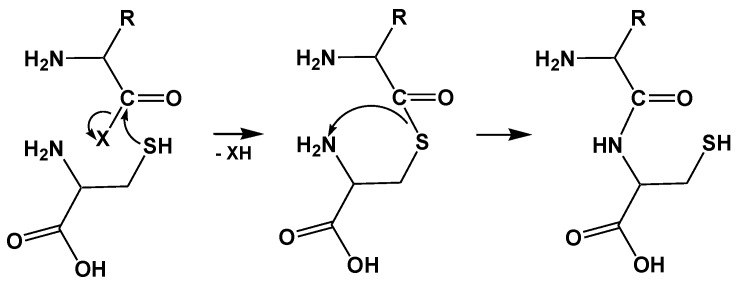
X = tRNA^Met^ or AMP; R = side chain of Met or other amino acid.

With Cys as a thiol substrate and with Met~tRNA or Met-AMP in the active site, MetRS catalyzes the aminoacylation of Cys thiol with Met, forming Met~*S*-Cys thioester, which then rearranges to MetCys dipeptide. Facile intramolecular reaction results from the favorable geometric arrangement of the α-NH_2_ of Cys with respect to the thioester bond in Met~*S*-Cys (Scheme 2). That Met~*S*-Cys thioester is an intermediate is supported by the finding that Met-*S*-mercaptopropionate thioester forms with 3-mercaptopropionic acid (an analogue of Cys missing the NH_2_ group) as a thiol substrate.

With Hcy as a thiol substrate, MetHcy dipeptide is formed by a similar mechanism [55]. CysGly dipeptide is also aminoacylated by MetRS forming MetCysGly tripetide [55].

Active site residues of MetRS that are important for catalysis of the synthetic reactions (Hcy~AMP, Met~AMP, and Met~tRNA formation) and Hcy editing (Hcy-thiolactone formation) are also important for the Met-thioester formation reactions.

That Met and thiols bind at different sites is supported by the non-competitive inhibition of the thioester formation reaction by Met, which affects the *k*_cat_ but not *K*_m_ for Cys [55].

### 4.2. IleRS, ValRS, LysRS

In addition to MetRS, other Hcy-editing AARSs have the ability to catalyze the synthesis of aminoacyl~*S*-thioesters and aminoacyl-Cys dipeptides (Table 3). For example, with CoA-SH as a thiol, substrate Class I IleRS [55,64] and ValRS [64], and Class II LysRS [64], catalyze the synthesis of Ile~*S*-CoA, Val~*S*-CoA, and Lys~*S*-CoA thioesters, respectively. With Cys as a thiol substrate, ValRS, IleRS, and LysRS catalyze the synthesis of ValCys [65], IleCys [65,66], LysCys [38] dipeptides, respectively (Scheme 2).

However, in contrast to their high selectivity in the tRNA aminoacylation reaction, AARSs exhibit relaxed amino acid selectivity in the CoA-SH aminoacylation reaction (Table 4). For example, IleRS aminoacylates CoA-SH with noncognate Val, Leu, Thr, Ala, and Ser in addition to the cognate isoleucine. The catalytic efficiencies for the non-cognate amino acids are only 10-fold (for Val) to 370-fold (for Thr) lower than the catalytic efficiency for the cognate isoleucine in the CoA-SH aminoacylation reaction. ValRS is even less selective and catalyzes aminoacylation of CoA-SH with threonine, alanine, serine, and isoleucine, in addition to valine (Table 4) with catalytic efficiencies only 2.4-fold (for Thr) to 28-fold (for Ile) lower than the catalytic efficiency for the cognate Val.

LysRS is the least selective and aminoacylates CoA-SH with noncognate leucine, threonine, alanine, valine, and isoleucine, in addition to the cognate lysine. *The rates of transfer of noncognate leucine and threonine are faster than the rate of lysine transfer to CoA-SH, while other noncognate amino acids are transferred by LysRS at rates only 1.1- to 8-fold slower*. The aminoacylation of CoA-SH with noncognate amino acids is prevented by the cognate amino acid, indicating that the ability to aminoacylate CoA-SH with a noncognate amino acid is an inherent property of IleRS, ValRS, and LysRS [64]. Apparently, binding of CoA-SH relaxes the selectivity of the amino acid substrate-binding site, whereas binding of tRNA increases the selectivity towards the cognate amino acid. The relaxed amino acid selectivity is indicative of a more primitive aminoacylation system involving CoA-SH that may have originated in a Thioester World (Section 9).

### 4.3. Arginyl-tRNA Synthease (ArgRS), Aspartyl-tRNA Synthetase (AspRS), Seryl-tRNA Synthetases (SerRS)

Surprisingly, AARSs that do not misactivate/edit Hcy also have the ability to catalyze the thiol aminoacylation reactions (Table 3). For example, Class I ArgRS [65] catalyzes aminoacylation of Cys, cysteamine, Hcy, and other thiols. With Cys or Hcy as thiol substrates ArgRS catalyzes the synthesis of ArgCys and ArgHcy dipeptides with Arg~*S*-Cys and Arg~*S*-Hcy thioesters as intermediates formed by a mechanism analogous to that depicted in Scheme 2 for MetRS. The rate enhancement by ArgRS of the Arg-thioester formation reaction with Cys is >3300-fold. Remarkably, the aminoacylation of Cys exhibits some degree of stereospecificity with a 6-fold preference for *l*-Cys vs. *d*-Cys. Further, ArgRS has the ability to add Arg to *N*-terminal Cys of Cys-Gly dipeptide forming ArgCysGly tripeptide [65].

Class II AspRS catalyzes aminoacylation of CoA-SH, pantetheine, Cys, and Hcy with Asp, forming Asp~*S*-CoA and Asp~*S*-pantetheine [63] thioesters, and AspCys [38,63] and AspHcy [38] dipeptides, respectively. Although other thiols are aminoacylated with Asp by AspRS, CoA-SH, pantetheine, and cysteine are the preferred substrates [63]. Similar to ArgRS [65], the AspRS-catalyzed aminoacylation of Cys is stereospecific, with a 10-fold preference for *l*-Cys vs. *d*-Cys.

Class II SerRS [63] catalyzes aminoacylation of Cys and other thiols. Aminoacylation of Cys catalyzed by SerRS leads to the formation of SerCys dipeptide with Ser~*S*-Cys thioester as an intermediate (Scheme 2). The rate enhancements by SerRS, AspRS, and LysRS of the aminoacyl thioester formation reaction are up to 30,000-fold [38]. Thus, the ability to aminoacylate CoA-SH, Cys, Hcy, and other thiols is a general feature of the catalytic domains of both Class I and Class II AARSs [10,11,62].

The thioester chemistry of AARSs underlying their ability to support synthesis of dipeptides, is reminiscent of the adenylation domains of non-ribosomal thio-template peptide synthetases [69,70], which belong to the ANL superfamily that also includes firefly luciferase and acyl-CoA synthetases, but is structurally unrelated to AARS families. However, as discussed in Section 5, Section 6 and Section 7 below, genomic comparisons reveal that AARSs are *structurally related* to enzymes participating in coenzyme A/sulfur metabolism and peptide bond synthesis, which has important evolutionary implications and sheds a light on the origin of coded peptide synthesis, discussed in Section 9.

## 5. Class I AARS Are Related to Proteins Involved in Sulfur/CoA Metabolism

The ribosomal translation apparatus is the most conserved component of cellular metabolism [1,37,71]. Its core components, including Class I and Class II AARSs and ribosomes, belong to the oldest fold families that can be traced back to the last universal common ancestor (LUCA) of life and were the earliest systems to evolve into a form close to that of the present day before the divergence of the three kingdoms of life from the LUCA [1,34,35,72]. Phylogenetic analyses of structural domains present in proteomes [34] and of substructures of RNA molecules [35] suggest that catalytic domains of AARSs have appeared before the ribosome [34,35]. The evolution of AARS from their respective common ancestors must have predated the LUCA [73,74].

**Figure 7:**
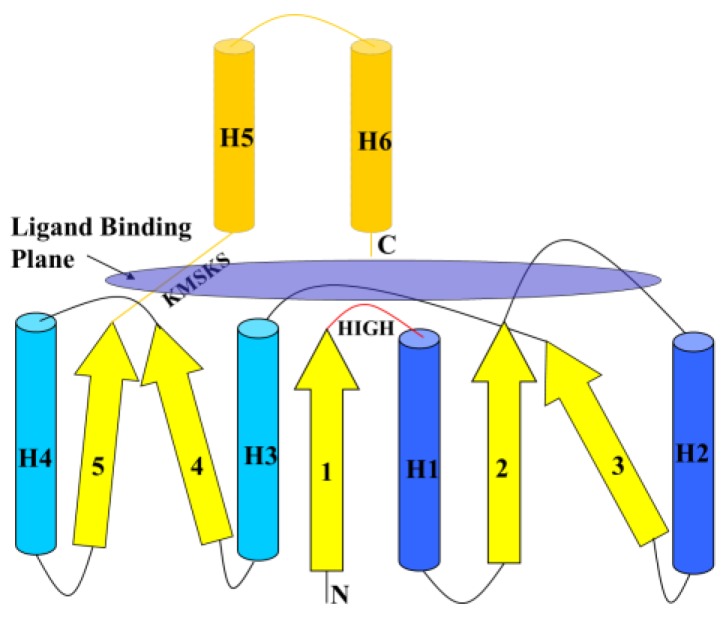
Topological display of the catalytic core of the HUP (HIGH-signature proteins, UspA, and PP-ATPase) domain proteins. Adapted with permission from ref. [73].

**Figure 8:**
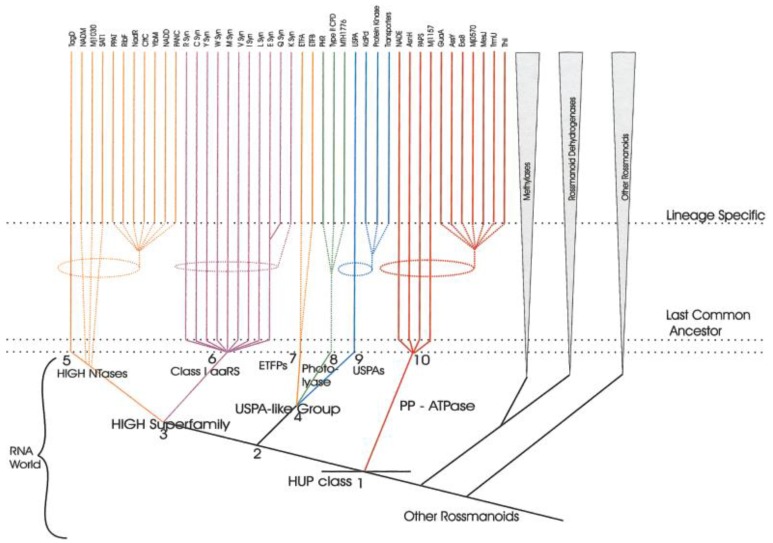
Evolutionary scenario (cladogram) for the HUP domain class (HIGH-signature proteins, UspA, and PP-ATPase). The specific characters associated with each of the nodes as derived through the cladistic analysis are as follows. **Node 1** (the HUP domain): presence of a core 5-strand sheet in the 3–2–1–4–5 order; **Node 2**: configuration of the region between the last helix and strand of the HUP domain, strand 4 and 5 hydrogen-bonded through most of their lengths; **Node 3**: HIGH motif in the loop between strand 1 and helix 1, bihelical extension *C*-terminal to the core HUP domain, with a secondary role in nucleotide-binding; **Node 4**: loop between strand 3 and helix 4 tends to face outward, helix 4 tends to be placed behind the central sheet, no strongly conserved motif between strand-1 and helix-1, possible loss of ability to hydrolyze α–β bond in ATP; **Node 5**: nucleotidyl transferase activity, two small residues in the KMSKS loop; **Node 6**: AARS activity, classical KMSKS motif; **Node 7**: insertion of a β-hairpin between helix 4 and strand 5; **Node 8**: fusion with large, α-helical *C*-terminal domain; **Node 9**: distinct loop between strand 4 and helix 4; **Node 10**: SXGXDS motif between strand-1 and helix-1, conserved helix *N*-terminal to strand 1. The conserved families and their probable temporal points of origin are shown for each of the six major lineages. Dotted ellipses and lines leading to a particular lineage indicate uncertainty regarding its emergence. Reproduced with permission from ref. [73].

The catalytic domain of Class I AARSs includes a three-layered α/β/α domain with five core β-strands in the order 3-2-1-4-5 surrounded by four α-helices (Figure 7). The signature HIGH motif is located in the loop between strand 1 and helix 1. Class I AARSs also have an extension that consists of a loop, containing the KMSKS motif, followed by two helices *C*-terminal to the α/β/α domain. The HIGH and KMSKS motifs are also present in a diverse family of nuclotidyltransferases. Genomic and structural comparisons show that catalytic domains of Class I AARSs and nucleotidyltransferases form a distinct HIGH superfamily that is related to two other superfamilies: PP-ATPases, a diverse superfamily of domains that catalyze reactions involving hydrolysis of the α-β pyrophosphate bond in ATP, and USPA-like group, that includes USPA domains, electron transport flavoproteins, and photolyases. Together these superfamilies comprise a distinct class of α/β/α domains designated the HUP (HIGH-signature proteins, UspA, and PP-ATPase) domain [73] (Figure 7). Similarities between these protein families were initially detected using structural comparisons but can also be detected at the sequence level. A structure-based multiple sequence alignment shows that specific features of the HUP domain include a core of five β-strand sheet in the 3-2-1-4-5 order and four α-helices, horizontal depression of β-strand 3 with respect to the rest of the sheet, crossover of β-strand 4 and 5 or their extensions, a sequence motif corresponding to β-strand 4 with a conserved small amino acid residue at its C-terminus, and ATP ligand (Figure 7). These features distinguish the HUP family from other domains with a Rossman-fold-like geometry (Figure 8) and suggest a monophyletic origin of the HUP domains [73].

In addition to Class I AARSs, the HIGH superfamily family includes protein families involved in CoA-SH biosynthesis such as panthotenate synthase (pantoate-β-alanine ligase) PanC [75], phosphopantetheine adenosyltransferase PPAT [76], and sulfate metabolism (sulfate adenylyltransferase SAT1) [73]. The HIGH superfamily is a distinct lineage within the HUP family (Figure 8), characterized by the HIGH motif between β-strand 1 and α-helix 1, and a two-helix extension at the C-terminus of the core domain via a loop with a KMSKS or related sequence involved in nucleotide binding (Figure 7). The second lysine of the KMSKS motif is almost always present in Class I AARSs but absent in other members of the HIGH superfamily, consistent with different modes of interaction with ATP phosphates.

An evolutionary link between Class I AARSs and enzymes of CoA metabolism is revealed by a structure-based alignment of amino acid sequences of PanC, PPAT, GluRS, GlnRS, and TyrRS, which shows that sequences corresponding to the HIGH and KMSKS signature motifs are present, although the KMSKS motif is only evident in structural alignment [75]. Serine at position three and basic or hydroxyl function at positions four and five of the KMSKS motif are conserved in all structures. The superposition of crystallographic structures of CoA-SH biosynthetic enzymes (pantothenate synthtase PanC, phosphopantetheine adenylyltransferase PPAT) and Class IAARSs (GluRS, GlnRS, TyrRS) shows that the highest ranking structural matches to the Cα coordinates of the *N*-terminal domain of *E. coli* PanC are the ATP-binding domains of *T. thermophilus* GluRS, GlnRS, and TyrRS, *E. coli* GlnRS, *B. stearothermphilus* TyrRS, and *E. coli* PPAT [75]. Although sequence identities are low (10.8% to 14.5%), these structures are very similar with a root mean square deviation (RMSD) of Cα atoms of 1.5 to 2.2 Å over 62–76 residues (Table 5).

Catalytic domains of class I AARSs are also similar to proteins involved in sulfate assimilation for cysteine biosynthesis such as sulfate adenosyltransferase [77] in the HIGH superfamily and 3′-phosphoadenosine-5′-phosphosulfate reductase [78] in the PP-LOOP superfamily [73]. These findings suggest an evolutionary link between class I AARSs and sulfur metabolism.

Analysis of patters of phyletic distribution of distinct families within these major lineages (Figure 8) suggests that the Last Universal Common Ancestor (LUCA) of modern life encoded 15–18 α/β ATPases and nucleotide-binding proteins of the HUP domain. This points to an extensive radiation of HUP domains before the LUCA, during which class I AARSs emerged at a later stage [73]. This also suggests that substantial evolutionary diversification of protein domains occurred well before the present-day version of protein-dependent translation apparatus was established, possibly in the Thioester World (Section 9).

## 6. AARSs Are Related to Proteins Involved in Peptide Bond Synthesis

### 6.1. Class I AARSs

Two peptide bond-forming systems related to Class I AARSs have been identified. The first system includes mycothiol synthase MshC, a Class I CysRS paralogue that functions independently of tRNA. MshC activates Cys with ATP, forming Cys~AMP, and then transfers the activated Cys to an amino group of glucosamine in the mycothiol biosynthetic pathway [79,80]. MshC shares 36.1% primary sequence identity to CysRS. Crystallographic structure of MshC is similar to that of CysRS and other Class I AARSs with the catalytic Rossman-fold domain of five-stranded parallel β-sheet surrounded by α-helices [81]. A superposition of MshC and CysRS structures, excluding the anticodon-binding domain of CysRS, shows an RMSD of 2.70 Å for overlapping α-carbon atoms [80].

The second system includes cyclic dipeptide synthetases (CDPs) [82], a group of enzymes belonging to the HUP superfamily of Rosmanoids folds, related to catalytic domains of Class I TyrRS and TrpRS. The CDPSs participate in biosynthesis of biologically active diketopiperazines such as albonoursin (Alb) in *Streptomyces noursei*, pulcherrimin in *Bacillus subtilis*, and mycocyclosin in *Mycobacterium tuberculosis* [82,83]. CDPs do not activate amino acids, but use aminoacyl~tRNAs, synthesized by canonical AARSs, to form cyclodipeptides. The synthesis of the cyclo(Phe-Leu) dipeptide, the first step of the albonoursin [cyclo(α,β-dehydroPhe-α,β-dehydroLeu)] biosynthetic pathway is catalyzed by AlbC, which transfers Phe from Phe~tRNA^Phe^ to the conserved Ser37. The Phe-AlbC intermediate reacts with Leu~tRNA^Leu^, forming a dipeptidyl-AlbC, which undergoes intramolecular cyclization to generate the cyclo(Phe-Leu) dipeptide [84]. AlbC can also incorporate Tyr and Met from the corresponding AA-tRNAs into cyclodipeptides [82,83].

In some actinomycetes (*Actinosynnema mirum* and *Streptomyces* sp. *AA4*), the AlbC-like CDP genes are associated with genes encoding an acyl-CoA ligase [85]. The acyl-CoA ligase family of enzymes activates carboxyl groups with ATP and then transfers them to CoA-SH, forming acyl~*S*-CoA thioesters. Thus, this neighborhood association suggests that these CDPS might use aminoacyl~*S*-CoA (generated by an acyl:CoA-SH ligase) as substrate for dipeptide synthesis, rather than aminoacyl~tRNAs.

Genomic comparisons also identified bacterial Class I MetRS paralogues [85] that are proposed to participate in the synthesis of a dipeptide through the condensation of Met~AMP with the α-NH_2_ group of a lysine derivative acetylated at the ε-NH_2_. These MetRS paralogues are often found in operons containing non-ribosomal peptide synthetases and acyl-CoA ligases, which could charge CoA-SH with amino acids for use by the peptide synthetases [85].

### 6.2. Class II AARSs

Two other peptide bond-forming systems related to Class II AARSs have also been identified. The first system uses aminoacyl~tRNA while the second uses aminoacyl~AMP for the peptide bond formation. The first system includes a SerRS paralogue VlmL while the second system includes biotin-protein ligase BirA, a SerRS paralogue; PoxA, a LysRS paralogue; and an AspRS/AsnRS paralogue AsnA.

VlmL is a dedicated SerRS that produces Ser~tRNA^Ser^ that is used by VlmA in transferring serine to isobutylhydroxylamine in biosynthesis of the antibiotic valanimycin by *Streptomyces viridifaciens* [86]. VlmL is essential and cannot be substituted by the canonical SerRS, suggesting that VlmL-VlmA complex formation is required for the VlmA function.

BirA is a protein ligase that attaches biotin to *N*ε-amino group of lysine residues of metabolic proteins involved in caboxylation or decarboxylation, e.g., acyl-CoA carboxylase [87]. Biotin is activated with ATP, forming BirA•biotin~AMP. The catalytic domain of BirA (residues 60–270) includes seven-stranded β-sheet and five α-helices with topology identical [87] to that observed in the catalytic domain of SerRS [88]. The RMSD for the superposition of 31 α-carbon atoms in the sheet of both structures is very low, 1.17 Å, indicating that the twist and curvature of the β-sheet are very similar. Two α-helices on sides of the sheet are also topologically equivalent and occur in the same sequence order. The binding sites for biotin in BirA and Ser~AMP analogue in SerRS occupy equivalent positions with respect to the β-sheet. Structural and functional similarities between BirA and SerRS suggest that their catalytic domains diverged from a common ancestor [87].

PoxA is a LysRS paralogue, the first known AARS paralogue that modifies a protein with an amino acid, transfers β-lysine to the ε-NH_2_ group of a conserved Lys34 residue of translation elongation factor P [89,90]. β-Lysine is first activated with ATP, forming β-Lys~AMP.

AsnA is an AspRS/AsnRS paralogue [91] that activates β-carboxyl of aspartic acid with ATP to form β-Asp~AMP and then transfers β-aspartate to ammonia forming asparagine.

## 7. Thioester Chemistry of SerRS Homologues from Methanogenic Archaea

Genome sequences of methanogenic archaea encode AA:CP ligases homologous to the catalytic domain of Class II SerRS [92], which aminoacylate the thiol of phosphopantetheine prosthetic group of a carrier protein (CP) with Gly or Ala, but do not aminoacylate tRNA. CP is encoded in the same operon that encodes the AA:CP ligase in the bacterial chromosome. These AA:CP ligases support the aminoacyl-thioester formation reaction similar to the CoA-SH/pantetheine aminoacylation reactions catalyzed by canonical AARSs (Scheme 1). Crystallographic studies show the pantetheine thiol arm enters the AA:CP ligase active site from the opposite direction relative to the entry of the 3′-end of tRNA in the canonical SerRS, a mode of interaction predicted for CoA-SH and tRNA within the active sites of AARSs. Further, similar to AARSs, AA:CP ligases catalyze aminoacylation of free CoA-SH and Cys with Ala and Gly, forming AA~*S*-CoA (Scheme 1) and AA-Cys dipeptides (Scheme 2), respectively [93], indicating that AA:CP ligases are both structurally *and* functionally related to canonical class II SerRS. These similarities suggest that catalytic domains of present-day AARSs may have evolved from ancestral forms that functioned as AA:CoA-SH ligases and represent molecular fossils that originate from an ancient catalytic domain that utilized thioester chemistry to activate amino acids for non-coded peptide bond synthesis, before acquiring the ability to aminoacylate tRNA. The AA:CoA-SH ligase activities of extant AARSs and CPs appear to be vestiges of an evolutionary link between thioester-dependent and RNA-dependent peptide synthesis [63,64].

## 8. Acyl~*S*-CoA Thioesters and Aminoacyl~tRNA Esters are Used in Peptide Bond Synthesis Catalyzed the Gcn5-related *N*-acetyltransferases (GNAT) Fold Enzymes

The Gcn5-related *N*-acetyltransferases (GNAT) are a superfamily of enzymes that are universally distributed in nature and use acyl~*S*-CoA to acylate their cognate substrates [94]. These include histone acetyltransferases, ribosomal protein S18 *N*α-acetyltransferase (RimI), protein *N*-myristoyltransferase, aminoglycoside *N*-acetyltransferases, serotonin *N*-acetyltransferase, glucosamine-6-phosphate *N*-acetyltransferase, mycothiol synthase (MshD), and the Fem family of amino acyl transferases. MshD catalyzes the final step in mycothiol biosynthesis, the acetylation of the α-amino group of cysteine attached to glucosamine part of mycothiol using acetyl~*S*-CoA as a substrate [95].

**Figure 9:**
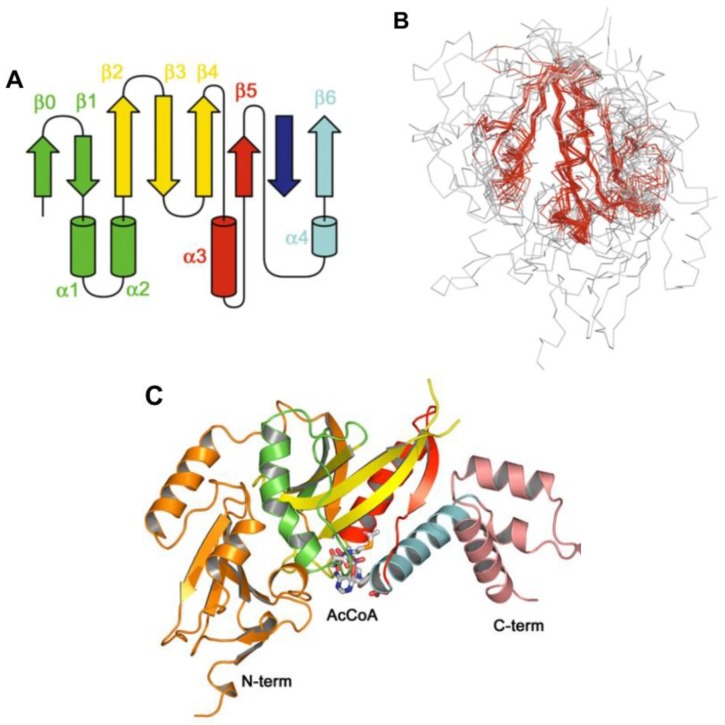
(**A**) Topology of the GNAT fold. From the *N*-terminus, secondary structural elements are colored green (β1, α1, α2), yellow (β2–4), red (α3, β5) and blue (α4, β6). The dark green (β0) *N*-terminal strand is not completely conserved and the deep blue *C*-terminal strand may be from the same monomer, or contributed by another; (**B**) Superposition of 15 GNAT structures. Residues in which the RMSD is <2.7 Å are highlighted in red; (**C**) Yeast Hat1 histone acetyltransferase in complex with Ac-*S*-CoA (1BOB.pdb). Reproduced with permission from ref. [94].

Crystallographic studies of the GNAT fold proteins with bound acetyl-*S*-CoA or CoA-SH show that the GNAT fold is a phosphopantetheine-binding domain [95], which contains a central five-β-stranded mixed polarity sheet with four α-helices (Figure 9) [94]. Two of the α-helices lie on top of the β-sheet aligned in parallel with the β-strands. The other two helices are stacked on the bottom of the β-sheet with one α-helix aligned in parallel with the β-strands and the other at a 60° angle. The GNAT fold has two distinct binding sites: one for the pyrophosphate moiety of CoA-SH with a signature motif Q/RxxGxG/A and another site for the pantetheine arm. The pyrophosphate-binding is located at a loop between β-strand 4 and α-helix 3, while the pantetheine arm-binding site is located between β-strands 4 and 5, which splay apart to allow the pantetheine arm to make pseudo β-sheet interactions with the exposed backbone atoms of β-strand 4 (Figure 9).

**Figure 10:**
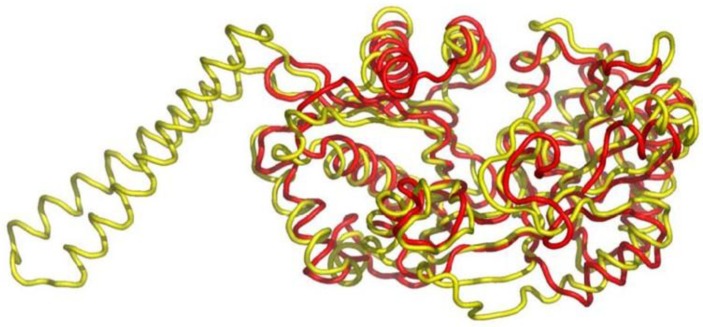
Superposition of FemX (red) with *S. aureus* FemA (yellow, PDB 1LRZ). The overall structures are similar: RMSD is 2.8 Å for the 309 Cα atoms). The major structural difference is the absence in FemX of the coiled-coil region constituted by two helices (residues 246–307; found in SerRS) inserted in FemA domain 2. Reproduced with permission from ref. [97].

However, the GNAT fold of some proteins binds tRNA, instead of CoA-SH [96,97]. For example, FemABX ligases of the GNAT fold (Figure 10) use aminoacyl~tRNAs as substrates for peptide ligation in the cell wall peptidoglycan synthesis in the Gram-positive bacteria. The Fem ligases catalyze the addition of l-amino acids and glycine from aminoacyl-tRNAs synthesized by AARSs to the ε-amino group of lysine in the pentapeptide (l-Ala-d-γ-Glu-l-Lys-d-Ala-d-Ala) of a peptidoglycan. Unfortunately, details of tRNA-GNAT fold interaction are not known, because the structures of any of those proteins in complexes with tRNA are not yet available.

**Figure 11:**
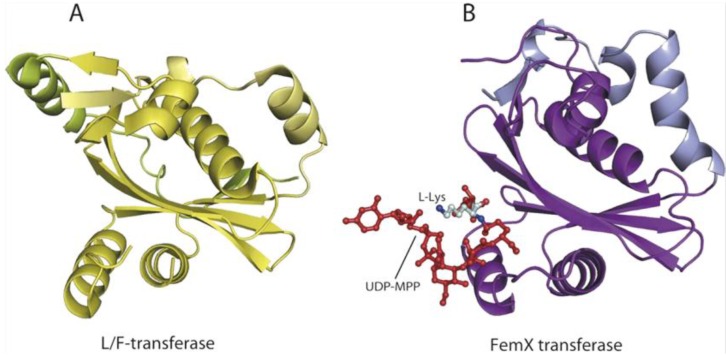
The GNAT superfamily fold in Leu/Phe-tRNA-protein transferase (L/F-transferase) (**A**) and FemX transferase (**B**). Uridine 5’-diphosphate-*N*-acetylmuramoylpentapeptide (UDP-MPP) bound in the FemX transferase structure is colored red and depicted as a ball-and-stick model. The l-Lys of UDP-MPP is shown in pale cyan. The two structures superimpose with RMSD of 2.7 Å. Reproduced with permission from ref. [99].

Another member of the FemABX family, Phe/Leu transferase, uses aminoacyl-tRNA to transfer these amino acids to the *N*-terminal amino acid of proteins, usually lysine or arginine [98,99]. A structure-based search shows that FemX and Phe/Leu transferases have similar structures (Figure 11).

Despite their low sequence identity, 12%, the *C*-terminal domain of Phe/Leu transferase has a core region, formed by six β-strands and four α-helices, found in the GNAT family proteins, which is involved in CoA-SH binding. *C*-terminal domains of Phe/Leu transferase and FemX superimpose with an RMSD of 2.7 Å for the conserved GNAT fold.

The GNAT superfamily proteins provide the second example of a protein fold, the first being the Rossman-fold in AARSs, which can evolve to bind both CoA-SH and tRNA.

## 9. Evolutionary Implications

As discussed in Section 4, Class I ArgRS and CysRS as well as class II AspRS and SerRS do not possess the Hcy editing function but nevertheless have the ability to catalyze the thiol aminoacylation reaction. This suggests that these AARSs possess a vestigial thiol-binding site, functionally similar to the thiol binding sites of Class I MetRS, ValRS, LeuRS, and IleRS. The ability of extant AARSs to catalyze the synthesis of aminoacyl thioesters and peptides suggests a vestigial thiol-dependent peptide synthesizing function, reminiscent of non-ribosomal peptide synthesis involving thioesters [69,70,100].

The AA:thiol ligase activity of the present-day AARSs suggests an evolutionary link between thioester-dependent and RNA-dependent peptide synthesis. Another example of a link between thioester-dependent and RNA-dependent peptide synthesis is provided by the GNAT fold superfamily of enzymes, discussed in Section 8. Taken together, these findings suggest that ancestral AARSs may have functioned as AA:pantetheine or AA:CoA-SH ligases before acquiring the ability to aminoacylate tRNA. CoA-SH itself appears to be a primitive analogue of tRNA [101] in that both molecules can carry amino acids for peptide bond synthesis [63]. Consistent with this scenario are the findings that IleRS, ValRS, and LysRS exhibit relaxed amino acid selectivity in the CoA-SH aminoacylation reaction, expected of a more primitive amino acid activating system in contrast to essentially absolute selectivity of IleRS and ValRS in the tRNA or RNA-minihelix aminoacylation reactions [64].

### 9.1. Prebiotic Synthesis of Amino Acids and Organosulfur Compounds

**Figure 12:**
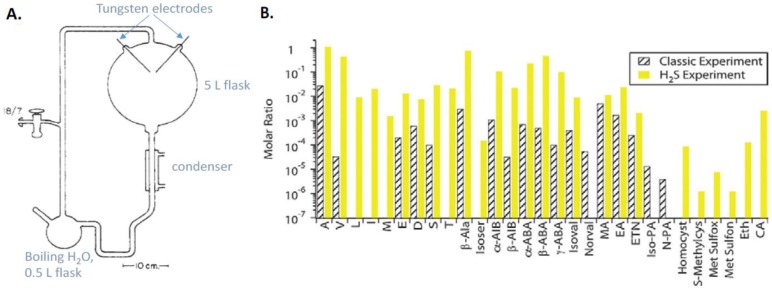
Amino acids and organosulfur compounds produced in the spark discharge experiments. (**A**) Diagram of the spark discharge apparatus (adapted from ref. [108]); (**B**) Abundances of amino acids and organosulfur compounds relative to glycine. Letter amino acid abbreviations are used. Reproduced from ref. [103] with permission.

Classical spark discharge experiments have established that amino acids are produced under possible primitive Earth conditions [102]. Organosulfur compounds are also formed under simulated prebiotic conditions [103] (Figure 12). For instance, methionine is formed on a simulated primitive Earth atmosphere containing methane, nitrogen, ammonia, water, and H_2_S or CH_3_SH subjected to spark discharges [104]. Cysteine forms in gas mixtures containing methane, ethane, ammonia, water, and hydrogen sulfide irradiated with long-wavelength ultraviolet light [105]. Amino acids can also form on pyrite or other metal sulfides under simulated volcanic conditions [106]. However, organic sulfur-containing amino acids are formed more readily in H_2_S-containing primitive atmospheres [107].

Acrolein is also a product of the spark discharge and is thought to be an intermediate in the prebiotic synthesis of methionine and Hcy [104], in addition to homoserine, glutamic acid and α,γ-diaminobutyric acid. Analysis of samples from an unreported 1958 Stanley Miller experiment shows that homocysteic acid, cysteamine, *S*-methyl-cysteine, ethionine, methionine sulfoxide and sulfone, in addition to methionine, form under prebiotic conditions [109]. Components of CoA-SH (β-alanine, pantoyl lactone cysteamine, and adenosine) are known to be likely prebiotic compounds. The pantetheine moiety of CoA-SH is obtained by mildly heating (40 °C) solutions of pantoyl lactone, β-alanine and cysteamine [110]. These findings support the suggestion that organosulfur compounds and thiols played important roles in prebiotic evolution [111].

**Figure 13:**
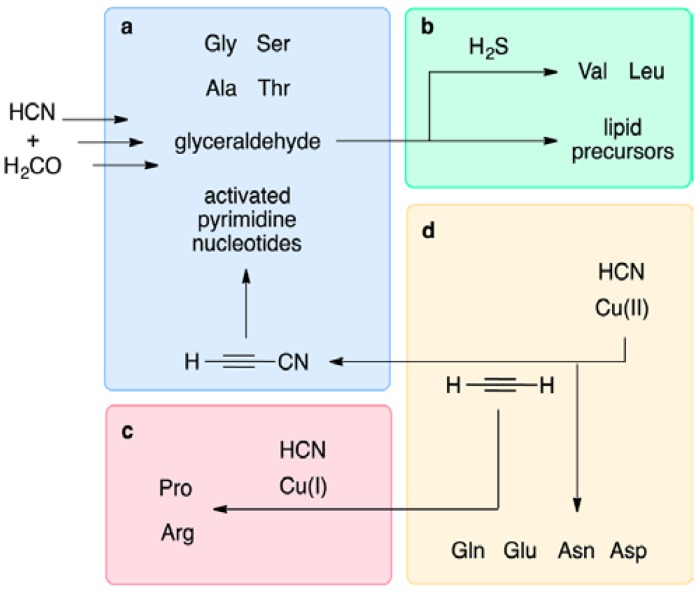
Prebiotic syntheses of amino acid, ribonucelotide, and lipid precursors. Reproduced from ref. [113] with permission.

Another approach to prebiotic chemistry, called “cyanosulfidic” chemical homologation, and relying on hydrogen cyanide as the sole carbon and nitrogen source, H_2_S as a reductant, and ultraviolet light and Cu(I)/Cu(II) catalysis of photo-redox cycling, can generate with high efficiency the building blocks of protein, RNA, and lipids [112] (Figure 13).

Reductive homologation of HCN (**a**) provides C2 and C3 sugar precursors of amino acids Gly, Ala, Ser, and Thr, as well as of ribonucleotides. Reductive homologation of the products of glyceraldehyde isomerization and reduction leads to lipid precursors, as well as amino acids Val and Leu (**b**). Cu(I) catalyzed cross-coupling followed by reductive homologation gives precursors of Pro and Arg (**c**), while Cu(II)-driven oxidative cross-coupling leads to precursors of Gln, Glu, Asn, and Asp (**d**) [112].

This simple approach inexorably leads to the very set of molecules used by modern biology. Different components may be delivered at different times and places via pools and streams, rainfall, and evaporite basins. Not only does this approach suggest that the set of proteinogenic amino acids might be preordained for life, it also overcomes perceived incompatibilities between the key subsystems and suggests that they could have developed together rather than sequentially [113].

### 9.2. The Thioester World

Sulfur is an important component of the present-day metabolic pathways. Several coenzymes, including CoA-SH and pantetheine, and two of the 20 proteinogenic amino acids, methionine and cysteine, contain sulfur. CoA-SH is thought to be a molecular fossil from an early metabolic state [114], “*a surviving representative of a group of thiol-amino acid-nucleotide compounds which contained within their structures the potential for both peptide synthesis and nucleotide extension*” and “*a primitive analogue of tRNA*” [101]. Extant AARSs exhibit a vestigial ability to catalyze aminoacylation of CoA-SH [64]. Both tRNA and CoA-SH, or its homologue pantetheine, can carry amino acids for peptide bond synthesis [63]. A more recent finding points to a close metabolic relationship between CoA-SH and RNA. For example, a chemical screen reveals a number of small molecule-RNA conjugates, including 3′-dephospho-CoA-SH and its acetyl, succinyl, and methylmalonyl-thioester derivatives [115]. These CoA species are attached at the 5′-terminus of small (<200 nucleotide) bacterial RNA that have yet to be identified. There are ~100 CoA-RNA molecules per *E. coli* cell [115], which suggests that these species are 10-fold less abundant than Phe-tRNA^Phe^ [116].

**Scheme 3:**
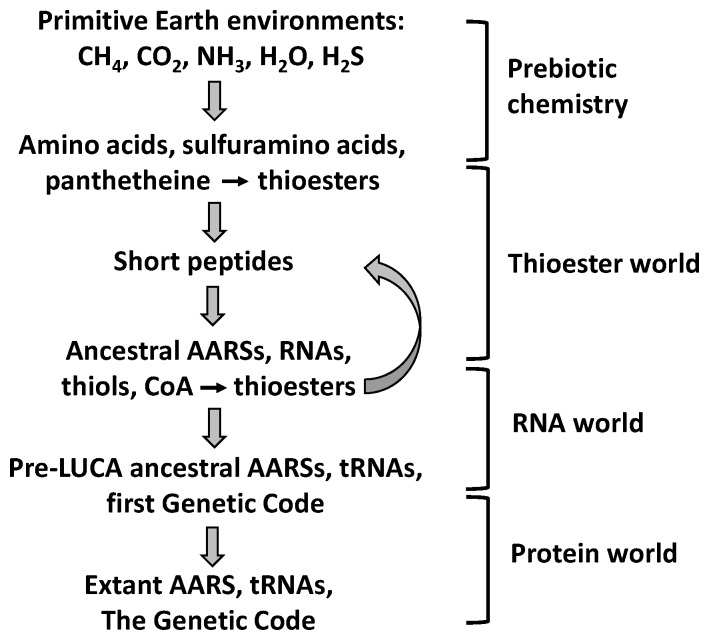
Major stages in the evolution of life.

Thioester chemistry underlies the participation of CoA-SH in acyl group activation in many biological processes in the three kingdoms of life. As the thioester bond is crucial in biochemical processes in extant organisms, then, by the principle of congruence, it must also have been important in the origin of life [101,111]. It is thought that CoA-thioesters originated very early in the development of life, dating back to the LUCA [117]. Particularly relevant in this context is the role of pantetheine, a precursor of CoA-SH, in the activation of amino acids for non-coded peptide synthesis, which forms the basis for Lipmann’s proposal that the thioester-dependent mechanism of peptide bond formation may have preceded the RNA-dependent mechanism of protein synthesis in the development of life [118]. This proposal was taken further by De Duve who pointed out that peptides form spontaneously from Aminoacyl Thioesters in aqueous solution [119] and suggested that Thioesters, which would have provided proto-metabolism with *catalysis* and *energy*, were among early organic molecules that seeded the development of life on the prebiotic Earth, i.e., in the Thioester World that preceded the RNA World [111] (Scheme 3).

Since the original proposal [111], new findings providing strong support to the Thioester World hypothesis have been generated [120]. Comparative genomic, structural, and biochemical studies revealed that *the present-day AARSs contain telltale traces pointing to a crucial role of sulfur and Thioesters in the origin of amino acid activation and peptide bond synthesis*. Hydrogen sulfide was a predominant form of sulfur in the prebiotic world and, as shown by the spark discharge experiments in a simulated prebiotic Earth environment (Figure 12), facilitated prebiotic formation of amino acids and a variety of organosulfur compounds [103], including *S*-methyl-Cys, homocysteic acid, methionine, Met-sulfoxide, Met-sulfone, ethionine (Eth), and cysteamine (CA) [107].

Because it forms easily by demethylation of methionine in the presence of iron or copper [121,122] or under acidic conditions [41], Hcy must also have been present in prebiotic environments, as well as the thioester Hcy-thiolactone, which forms easily from Hcy under acidic conditions [123]. Due to its high chemical reactivity towards amine nucleophiles, Hcy-thiolactone could have participated in the peptide bond formation [40]. Although amino acids can form under simulated hot volcanic conditions in an alkaline aqueous Ni(OH)_2_/KCN/CO system [124], organosulfur compounds such as cysteine [105], homocysteic acid, cysteamine, and methionine are produced more readily from H_2_S-containing primitive atmospheres [107]. Pantetheine, a precursor to CoA-SH, is also formed under simulated prebiotic conditions [110].

Thioesters form easily under acidic conditions [41] and can spontaneously react with amino acids to form peptides [119]. The thioester bond [125] is a high-energy bond and thus is highly reactive towards amino groups [39,41]. Peptides can also form in other plausible prebiotic environments containing carbon monoxide, H_2_S or CH_3_SH on (Fe,Ni)S or pyrite FeS_2_ surfaces [126], or by using a plausible prebiotic condensing agent cyanamide [127]. However, peptide synthesis from thioesters is much more robust [119,128] than from pyrite/methyl sulfide- or cyanamide-dependent reactions.

The thioester chemistry of extant Class I and Class II AARSs (Table 4), and structural/ functional similarities of their catalytic domains to enzymes participating in CoA-SH and sulfur metabolism point to the origin of the amino acid activation and peptide bond synthesis functions in the Thioester World (Scheme 3). These findings strongly suggest that *before the development of coded protein synthesis, catalytic domains of ancestral AARSs may have facilitated the formation of aminoacyl thioestersupported non-coded peptide synthesis*. With Cys and Hcy as thiol substrates, AARSs support the synthesis of AA-Cys and AA-Hcy dipeptides. With Cys-Gly as a thiol substrate tripeptide AA-Cys-Gly forms in reactions catalyzed by AARSs.

Peptides containing *N*-terminal Cys can be extended to longer peptides by condensing with aminoacyl-thioesters in reactions analogous to native chemical ligation exploited in chemical synthesis of proteins [128]. Hcy-(AA)_n_ peptides are easily formed in reactions of Hcy~thiolactone with amino acids or dipeptides [39]. The α/β/α-layered and antiparallel β-sheet structures that gave rise to catalytic domains of primordial Class I and Class II AARSs, respectively, may have been assembled from short peptide hairpins [35,37] formed in non-coded thioester-dependent peptide synthesis. Cys-containing peptides must have played an important role in peptide -> protein evolution: Cys residues endow extant proteins with the ability to modify their structure by splicing out specific peptides [129].

With the advent of RNA, which may have been synthesized in catalytic pockets of ancestral AARSs [35] (as suggested by the ability of extant AARSs to catalyze the formation of AppppA and related dinucleoside oligophosphates [4,5]), the function of thiols (CoA-SH, panthethiene) in the non-coded peptide synthesis was expanded to RNA minihelices [64,130]. Before the emergence of the Genetic Code, ancestral AARSs could have facilitated formation of aminoacyl-S-CoA/panthetheine thioesters and aminoacyl-RNA esters for non-coded peptide assembly [63]. The thioester-dependent and RNA-dependent peptide synthesizing systems could have been developing in parallel, possibly via interrelated stages. Vestiges of this stage of prebiotic evolution, i.e., CoA-SH-dependent and RNA-dependent peptide synthesizing activities, are still visible in catalytic domains of extant AARSs [63,64] and the CDPs [82], respectively. The traces of the transition from the Thioester World to RNA World can also be found in the GNAT fold superfamily, which can use acyl~*S*-CoA thioesters or aminoacyl~tRNA esters as substrates for amide or peptide bond synthesis.

## 10. Conclusions

- Coded peptide synthesis has been preceded by a prebiotic stage, a Thioester World, in which thioesters played key roles.
- Remnants of the Thioester World can be found in extant aminoacyl-tRNA synthetases (AARSs) and related proteins:
- A thiol-binding site at the catalytic domain of AARSs confers the amino acid:thiol ligase activity;
- AARSs are structurally related to proteins involved in sulfur/CoA-SH metabolism and peptide bond synthesis;
- Protein folds that bind pantetheine/CoA-SH can also bind tRNA.

## Figures and Tables

**Table 1:** Average distances calculated from molecular dynamics simulation for Hcy~AMP bound in the active site of Class I AARSs.

	Average Distance, Å			
	MetRS	LeuRS	ValRS	IleRS
S_Hcy_···O_Asp/Glu_	5.30	5.51	4.94	4.70
S_Hcy_···C_carb_	3.91	5.19	4.14	4.66
C_carb_···O_Asp/Glu_	4.25	4.39	5.26	4.10
S_Hcy_···O_phos_	6.99	7.78	7.01	4.25

**Table 2:** Translational and post-translational incorporation of Hcy into HUVEC protein [60,62].

Cell Labeling Conditions	Translational		Post-Translational
	[^35^S]Hcy-Protein	[^35^S]Met-Protein	*εN*-[^35^S]Hcy-Lys-Protein
	%	%	%
[^35^S]Hcy (10 μM, 50 μCi/mL)	37	25	38
[^35^S]Hcy + folic acid, 10 μM	<1	>98	<1
[^35^S]Hcy + HDL, 1 mg/mL	68	25	7
[^35^S]Hcy + Met, 20 μM	12	76	12
Control, *εN*-[^35^S]Hcy-Lys-protein	<4	0	>96

**Table 3:** Hcy editing, thiol aminoacylation, and peptide synthesis by AARSs [10,11].

AARS	Hcy Editing	Thiol-Binding Site Residue	Thiol Aminoacylation	Peptide Synthesis	References
Class I					
MetRS	Yes	Asp359	Yes	Yes	[24,25,26,27,47,55,56,58,63,67]
LeuRS	Yes	Glu532	?	?	[25,26,58]
IleRS	Yes	Glu550	Yes	Yes	[25,26,58,63,64,66]
ValRS	Yes	Asp490	Yes	Yes	[58,64,65]
CysRS	No	?	Yes	?	[68]
ArgRS	No	?	Yes	Yes	[65]
Class II					
SerRS	No	?	Yes	Yes	[38,63]
AspRS	No	?	Yes	Yes	[38,63]
LysRS	Yes	?	Yes	Yes	[29,38]

?—Not examined.

**Table 4:** Class I IleRS and ValRS, and Class II LysRS exhibit relaxed amino acid selectivity in the CoA-SH aminoacylation reaction.

Amino Acid	IleRS, *k_cat_/K*_M_ (min^−1^·M^−1^)	ValRS, *k_cat_/K*_M_ (min^−1^·M^−1^)	LysRS, v (µM/h)
Isoleucine	440,000	115	0.24
Valine	45,000	3300	0.67
Leucine	7700	<5	2.7
Threonine	1200	1400	2.7
Serine		170	<0.1
Alanine	1300	500	1.7
Lysine			1.9

**Table 5:** Structural similarity of pantothenate synthetase Pan C to Class I AARSs and PPAT [75].

	GlnRS	GluRS	TyrRS	PPAT
Sequence identity to Pan C (%) *	10.8	14.5	11.2	14.5
RMSD of Cα atoms (Å)	1.8	1.5	2.2	1.8
Number of Cα aligned for RMSD	65	62	68	76

* Calculated for those residues used for RMSD calculation.
